# Preoperative Risk-Stratification of High-Risk Prostate Cancer: A Multicenter Analysis

**DOI:** 10.3389/fonc.2020.00246

**Published:** 2020-03-06

**Authors:** Brecht Chys, Gaëtan Devos, Wouter Everaerts, Maarten Albersen, Lisa Moris, Frank Claessens, Gert De Meerleer, Karin Haustermans, Alberto Briganti, Piotr Chlosta, Paolo Gontero, Markus Graefen, Christian Gratzke, R. Jeffrey Karnes, Burkhard Kneitz, Giansilvio Marchioro, Rafael Sanchez Salas, Martin Spahn, Bertrand Tombal, Henk Van Der Poel, Jochen Walz, Hendrik Van Poppel, Steven Joniau

**Affiliations:** ^1^Urology, Department of Development and Regeneration, University Hospitals Leuven, Leuven, Belgium; ^2^Laboratory of Molecular Endocrinology, KU Leuven, Leuven, Belgium; ^3^Department of Radiation Oncology, University Hospitals Leuven, Leuven, Belgium; ^4^Department of Urology, San Raffaele Hospital, University Vita Salute, Milan, Italy; ^5^Department of Urology, Jagiellonian University Medical College, Kraków, Poland; ^6^Department of Urology, University of Turin, Turin, Italy; ^7^Department of Urology, Martini Klinik am UKE GmbH, Hamburg, Germany; ^8^Urologische Klinik Und Poliklinik, Ludwig Maximilians Universität, Munich, Germany; ^9^Department of Urology, Mayo Clinic, Rochester, NY, United States; ^10^Department of Urology and Pediatric Urology, University Hospital Würzburg, Würzburg, Germany; ^11^Department of Urology, University of Piemonte Orientale, Novara, Italy; ^12^Department of Urology, Institut Mutualiste Montsouris and Paris Descartes University, Paris, France; ^13^Department of Urology, University Hospital Bern, Inselspital, Switzerland; ^14^Department of Urology, Cliniques Universitaires Saint Luc, Brussels, Belgium; ^15^Department of Urology, Netherlands Cancer Institute, Amsterdam, Netherlands; ^16^Department of Urology, Institut Paoli Calmettes Cancer Centre, Marseille, France

**Keywords:** prostate, prostate cancer, EMPACT, risk stratification, high risk prostate cancer

## Abstract

**Background:** Cancer-specific survival (CSS) within high-risk non-metastatic prostate cancer varies dramatically. It is likely that within this heterogenous population there are subgroup(s) at extraordinary risk, burdened with an exaptational poor prognosis. Establishing the characteristics of these group(s) would have significant clinical implications since high quality preoperative risk stratification remains the cornerstone of therapeutic decision making to date.

**Objective:** To stratify high-risk prostate cancer based on preoperative characteristics and evaluate cancer specific survival after radical prostatectomy.

**Method:** The EMPaCT multi-center database offers an international population of non-metastatic high-risk prostate cancer. Preoperative characteristics such as age, biopsy Gleason score, PSA and clinical stage were subcategorized. A multivariate analysis was performed using predictors showing significant survival heterogeneity after stratification, as observed by a univariate analysis. Based upon the hazard ratios of this multivariate analysis, a proportional score system was created. The most ideal group distribution was evaluated trough different score cut-off's. The predictive value was tested by the herald C index.

**Results:** An overall 5-years CSS of 94% was noted within the entire high-risk cohort (*n* = 4,879). Except for age, all preoperative risk factors showed a significantly differing CSS. Multivariate analysis indicated, T4 stage as being the strongest predictor of CSS (HR: 3.31), followed by ISUP grade 5 group (HR 3,05). A score system was created by doubling the hazard ratios of this multivariate analysis and rounding off to the nearest complete number. Multivariate analysis suggested 0, 4, 8, and 12 pts as being the most optimal group distribution (*p*-value: 0.0015). Five-years CSS of these groups were 97, 93, 87, and 70%, respectively. The calculated Herald C-index of the model was 0.77.

**Conclusion:** An easy-to-use pre-operative model for risk stratification of newly diagnosed high-risk prostate cancer is presented. The heterogeneous CSS of high-risk non-metastatic prostate cancer after radical prostatectomy is illustrated. The model is clinically accessible through an online calculator, presenting cancer specific survival based on individualized patient characteristics.

## Introduction

Prostate cancer (PCa) is the second most common cancer among men. It represents the 5th most frequent cause of cancer related death (1). According to the WHO cancer report (2014), 1.1 million men received a new diagnosis of prostate cancer in 2012 causing 0.3 million disease related deaths (2). Since the introduction of PSA screening in the beginning of the 80's an impressive incidence rise has been observed. Fortunately, this trend was counterbalanced by a reduction in mortality since the 90's due to earlier detection and improved curative treatments. Nevertheless, mortality attributed to PCa is expected to rise in the following decades implying an expanding burden to society (3).

Non-metastatic PCa is prognostically stratified as low, intermediate or high-risk as suggested by D'Amico in 1998 (4, 5). Currently, management of non-metastatic prostate cancer includes active surveillance, radical prostatectomy (RP) with or without pelvic node dissection and radiotherapy (RT) with or without androgen deprivation therapy (ADT). As illustrated by the PROTECT-Trial, no significant difference in low to intermediate risk Pca specific mortality was observed between RP and RT over a 10-years period (6). However, PCa specific mortality was low. Although low risk prostate cancer is most prevalent and known to have a good prognosis, high risk prostate cancer is less frequent but contributes most to PCa specific death (6).

Depending on fitness, low risk PCa is manageable trough active surveillance or radical prostatectomy (RP) without lymph node dissection (LAD). RP has shown to significantly reduce the overall mortality of Intermediate-risk prostate cancer (IRPCa) (7). If probability of lymph node invasion exceeds 5%, an additional extended LAD is recommended (4). Although general consensus concerning treatment of high-risk PCa is lacking, a multimodal strategy including RP with extended LAD is accepted by our in-house protocol (4).

High-risk PCa, according to the national comprehensive cancer network (NCCN), is defined as Gleason score ≥8, PSA > 20 ng/ml or clinical stage ≥T3a (8). Interestingly the EAU differs from this as it defines high-risk PCa starting at a T2c clinical stage (4). An overall established definition of high-risk disease is thus lacking. Remarkably, metastasis free survival (MFS) varies from 70 to 95% and 10-years biochemical recurrence (BCR) shows a variability of 50% (5, 9). Efforts to dissect this heterogeneity have been undertaken, as illustrated by Joniau et al. (10).

High quality risk stratification remains the cornerstone of therapeutic decision making. This retrospective study aims to stratify non-metastatic PCa into subgroups showing significantly differing CSS. Through this stratification, we aim to identify and correlate patient and tumor related characteristics so individual patients can be profiled within the heterogeneous group of non-metastatic high-risk PCa.

## Patients and Methods

### Patient Population

**Figure 1:**
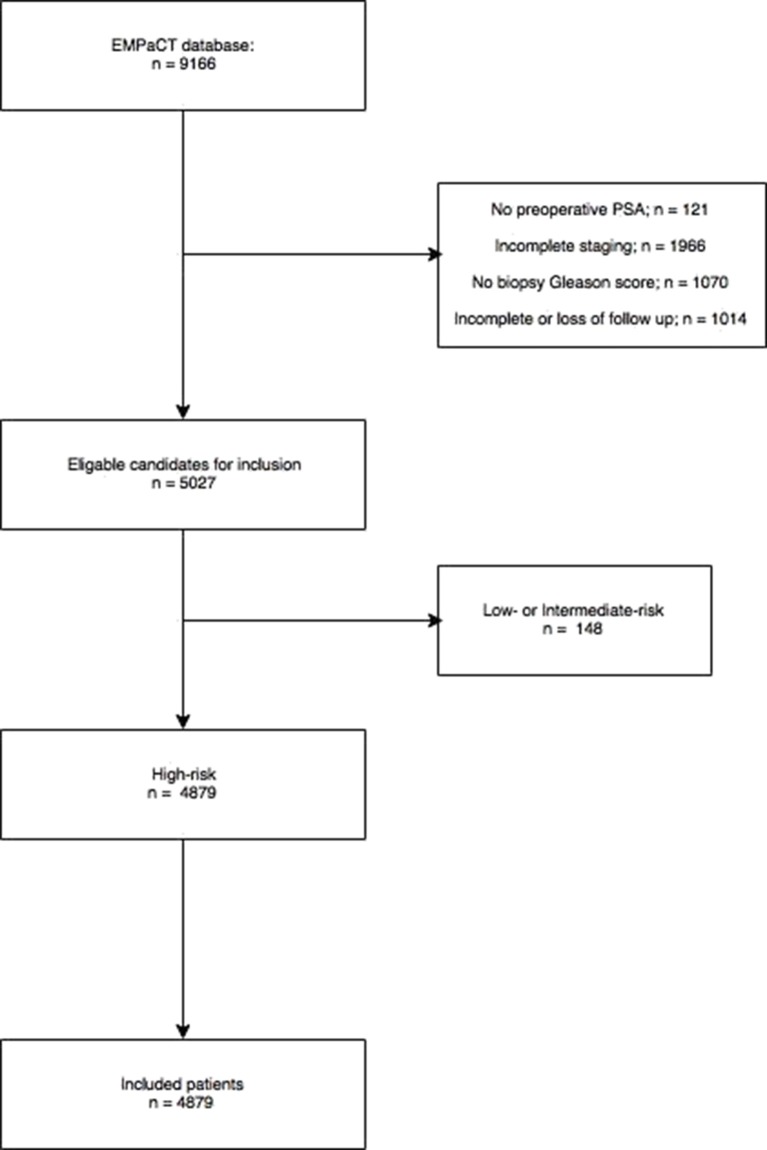
Patient selection EMPaCT database.

The European Multicenter Prostate Cancer Clinical and Translational (EMPaCT) research database served as the source for our patient cohort. This International research database contains 9,167 men from 14 institutions who underwent radical prostatectomy for non-metastatic high-risk PCa between 1986 and 2016. Each institution acted in accordance of their own standards, indications and treatment protocols. Since only patients with complete datasets could be included, the criteria for exclusion were defined as: lacking a preoperative PSA (*n*:121), absent Gleason biopsy score (*n*:1,070), incomplete staging (*n*:1,966) and lost to follow up (*n*:1,014). Staging was in accordance with the 2002 TNM system. All biopsies were evaluated by an experienced pathologist in each respective center. Follow up was defined as an annual clinical control with serum PSA measurement. Cancer related deaths were judged by the treating urologist or oncologist. Adjuvant and salvage therapies were admitted on individual bases and institutional preferences. From this eligible cohort, all high-risk (PSA ≥ 20 ng/ml and/or GS ≥ 8 and/or cT ≥ T2c) patients were identified and included (Figure 1).

### Statistical Analysis

Preoperative prognostic variables were identified and stratified into subcategories. PSA was subcategorized into a <20 ng/ml, a 20 ng/ml−50 ng/ml and a >50 ng/ml. Clinical stage was divided into T1, T2, T3a, and T3b+T4 categories. Biopsies were categorized by the international Society of Urological Pathology (ISUP) grading. Finally, a primary Gleason grade 5 subcategory was created and age was stratified into <60, 60–69, and ≥70 years old. A univariate analysis of these preoperative variables was performed to evaluate their impact on CSS. A multivariate cox regression analysis was performed using the significant variables. Based on the hazard ratio's (HR), a proportional score system was created. Multiple cut-off values were tried and different possibilities were compared by multivariate cox regression analysis. The most appropriate model was selected and its prognostic value was calculated using the concordance index (C-index). All univariate and multivariate analyses were performed using MedCalc Statistical Software version 17.9.7 (MedCalc Software bvba, Ostend, Belgium). The C-index was calculated using SAS-software version 9.4 (SAS Institute Inc., Cary, NC, USA). *P* < 0.05 were considered to be significant.

## Results

### Study Population

Four thousand eight hundred seventy-nine men met the criteria for final inclusion. A mean follow-up of 60.5 months was noted with an interquartile range of 65 months (21–84 months). The mean PSA amounted to 22.7 ng/ml (0–1,710 ng/ml). A biopsy Gleason Score of <7 was most prominent within HRPC. Clinical stage cT3 showed most prevalent, constituting 37.5% of all high-risk cases. An overview of the characteristic of the population is given in Table 1. The 5, 10, and 15-years CSS were 94, 89.5, and 84.6%, respectively.

**Table 1:** Characteristics of study population.

**Included patient cohort characteristics** ***n*** **= 4,879**				
Age	Mean (SD)		64.9 (6.8)	
	Median (IQR)		65 (60–70)	
PSA (ng/ml)	Mean (SD)		22.7 (41.6)	
	Median (IQR)		13 (7–27)	
	<20 μg/l		2,859 (58.6)	
	20–50 μg/l		1,415 (29)	
	>50 μg/l		373 (7.6)	
Clinical stage, *n* (%)	cT1		1,755 (36)	
	cT2		1,245 (25.5)	
	cT3	N.O.S.	1,830 (37.5)	745 (15.3)
		cT3a		948 (19.4)
		cT3b		137 (2.8)
	cT4		49 (1.0)	
biopsy Gleason score, *n* (%)	≤7		2,457 (50.4)	
	8	N.O.S	1,466 (30.0)	143 (2.9)
		3 + 5		156 (3.2)
		4 + 4		1,116 (22.9)
		5 + 3		51 (1.0)
	9	N.O.S.	855 (17.5)	60 (1.2)
		4 + 5		608 (12.5)
		5 + 4		187 (3.8)
	10		101 (2.1)	
Follow up (months)	Mean		60.5 (53.8)	
	Median		48 (21–84)	
	min		0	
	max		293	

*N.O.S, not otherwise specified*.

### Univariate Analysis

**Figure 2:**
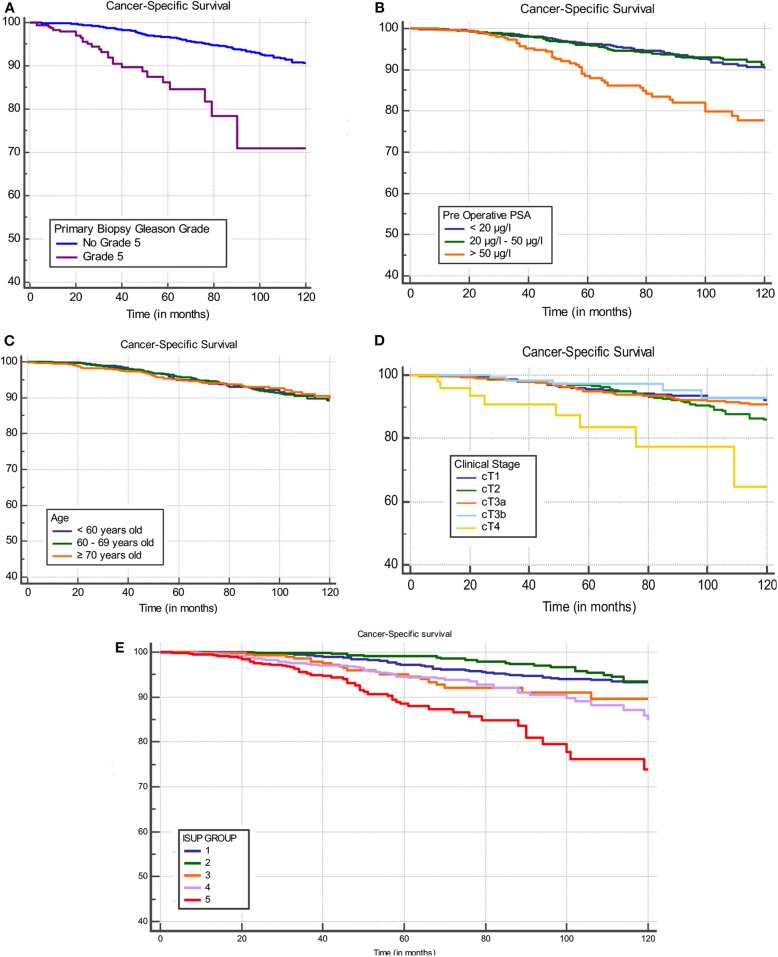
Univariate analysis of stratified preoperative risk factors: presence of a biopsy Gleoson grade 5 **(A)**, PSA **(B)**, age **(C)**, clinical stage **(D)**, and ISUP group **(E)**.

PSA, clinical stage, Gleason biopsy score, age and the presence of a Gleason grade 5 underwent categorization and univariate analysis for cancer specific survival as primary outcome (Figure 2). PSA was divided into three groups: <20, 20–50, and more than 50 ng/ml. Gleason score was categorized by the ISUP groups. Clinical stage was divided into four groups: T1, T2, T3a+b, and T4. Three age groups were identified by cut-off values of 60 and 70 years old. Finally, the presence of a primary Gleason grade 5 was dichotomised as present or absent. Except for age, all subdivisions of these preoperative risk factors showed significantly differing CSS.

### Multivariate Analysis

The preoperative risk factor groups which showed a significantly differing CSS were included in a multivariate cox regression analysis (Table 2). Biopsy characteristics were clearly the strongest CSS predictor. The presence of a primary Gleason grade 5 (HR: 7.17), followed by ISUP grade group 5 (HR: 3.05).

**Table 2:** Multivariate analysis of preoperative risk factors.

		***P*-value**	**Exp(b)**	**Points**
PSA	<20 ng/ml	Reference		0
	20–50 ng/ml	**0.03**	1.43	3
	>50 ng/ml	**<0.0001**	2.81	6
Clinical stage	≤T3b	Reference		≤T3b
	T4	**0.01**	2.73	6
ISUP	≤3	Reference		0
	4	**0.0001**	2.21	4
	5	**<0.0001**	3.05	6
	Any ISUP with primary grade 5	**<0.0001**	7.17	14

*Bold values statistically significant P < 0.05*.

Multiple combinations of clinical staging were tried in the multivariate analysis. However, only T4 showed a significantly different CSS as is illustrated in Figure 2D.

**Figure 3:**
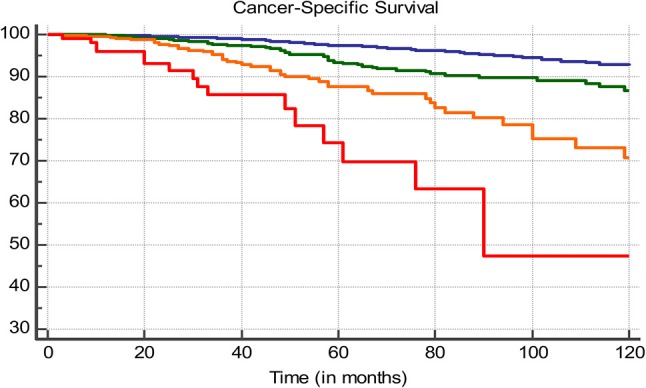
Ten-years CSS, blue line: 0–4 pts, green line: 5–8 pts, orange line 9–12 pts, red line: >12 pts.

Based upon the hazard ratios from the multivariate analysis, a proportional score was determined for each subgroup. This score system was then applied to all patients. Score-based groups were identified who showed significant differing CSS. Different cut-offs were evaluated. After multivariate analysis the 0–4, 5–8, 9–12, and >12 pts was selected as being the optimal distribution due to strongly differing CSS between all groups (*p* < 0.0001) (Figure 3, Table 3). Five-years CSS of these groups were 97.4, 92.8, 85.2, and 72.2%, respectively.

**Table 3:** Ten-years CSS, blue line: 0–4 pts, green line: 5–8 pts, orange line 9–12 pts, red line: >12 pts.

	**Number at risk**						
	**0 months**	**20 months**	**40 months**	**60 months**	**80 months**	**100 months**	**120 months**
0–4 pts 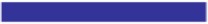	3,301	2,646	1,991	1,473	1,059	731	529
5–8 pts 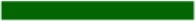	906	644	434	279	189	114	86
9–12 pts 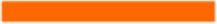	518	345	196	106	59	33	16
>12 pts 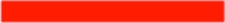	104	55	32	15	8	4	4

### Score Validation

In order to assess the predictive value of this score system, the concordance index (c-index) was determined. A value of 0.77 was noted, implying a good correlation between the model determined subgroups and the CSS. The model is accessible online through as an easy-to-use clinical tool. (https://app.calculoid.com/?#/calculator/41236).

## Discussion

**Figure 4:**
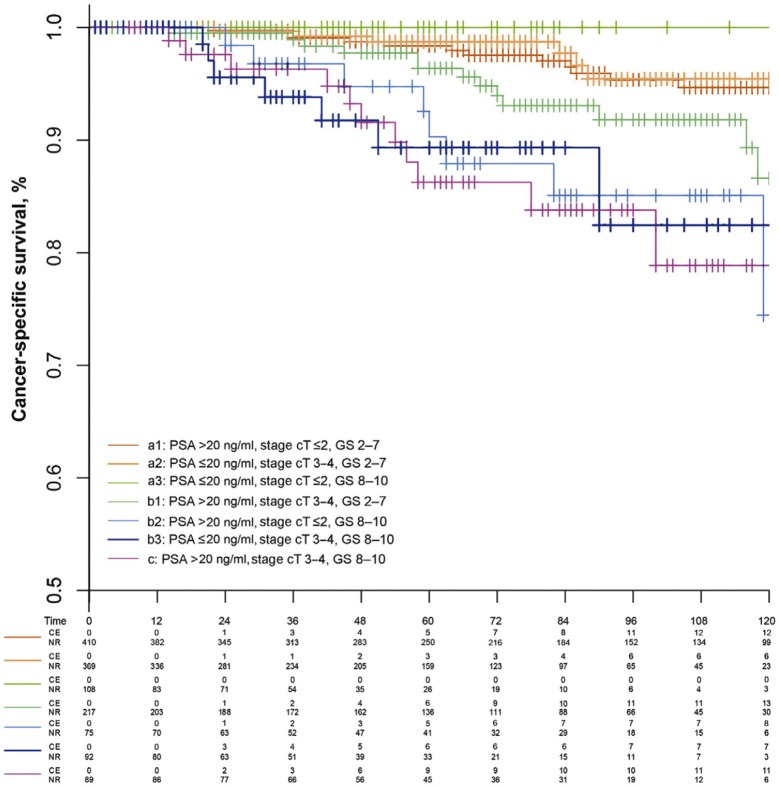
Prostate cancer–specific survival for the extended model with seven subgroups of high-risk prostate cancer patients (10).

When confronted with a new diagnosis of non-metastatic prostate cancer, it is common to divide patients into low-, intermediate- and high-risk subgroups (4). These groups are known to harbor a significantly differing prognosis. To date, this risk stratification remains the cornerstone of therapeutic decision making. Although there is no discussion concerning the need for surgical treatment in the high risk group, CSS is known to vary strongly thus suggesting this group to be quite heterogeneous (5). This can easily be illustrated by observing CSS after categorization by the number of high-risk factors. Intuitively a poorer prognosis is observed in patients showing multiple high-risk factors (Figure 4).

Historically, tumors with unfavorable characteristics (PSA > 100 ng/ml, Gleason Score 9–10, T4 or cN1) were not considered ideal candidates for surgery (8). This is largely due to fear for occult metastasis, which is not yet detectable by conventional staging technology at a given time. However, favorable results have been achieved in surgical treatment for non-metastatic, hormonal sensitive locally advanced prostate cancer (11).

Previous efforts have been undertaken to stratify high-risk PCa (9, 10). The capability to distinguish good from poor surgical candidates remains critical in clinical practice.

Sundi et al. illustrated that the presence of a primary grade 5 on biopsy, or ≥5 cores showing a Gleason score 8–10 were predictive for a significantly increased risk of metastasis and cancer specific mortality (5). Unfortunately, no data concerning number of positive biopsy cores is available in the EMPaCT database. However, univariate analysis of ISUP grading and presence of a primary Gleason grade 5 clearly shows its independent and strong prognostic significance. Thus, our findings are similar to Sundi et al.'s observation.

It has been suggested that PSA is a less valuable predictor (10, 12). Gontero et al. illustrated that, although prognosis diminishes with rising PSA, no absolute upper limit for radical prostatectomy exists (12). This biomarker is susceptible to a couple of difficulties. Firstly, it is a continuous variable. A clear cut-off is lacking. Secondly, our results suggest that PSA harbors the weakest CSS prognostic predictive value (HR 1.48). Only very high PSA values (>50 ng/ml) are good predictors for poor CSS (HR 2.97). These findings thus align with general belief that this biochemical marker should not be decisive in therapeutic decision making, except if extremely elevated.

Although age showed no independent value in the univariate analysis of CSS, it is very important in therapeutic decision making since age is mostly inversely proportional to general fitness. unfortunately, our data and model has no eye for comorbidity such as a Charlson score since this information was only available for a minority of patients.

Further evaluation shows that higher scoring patients were more likely to need adjuvant therapy such as androgen deprivation therapy, radiotherapy or both. Furthermore, we were able to illustrate that this score system proportionately correlates with positive surgical margins and lymph node invasion (Table 4).

**Table 4:** Need for (neo)adjuvant therapy, positive surgical margins, and lymph node invasion.

	***n***	**Neoadjuvant therapy (ADT)**		**Adjuvant therapy (ADT/ADT+RT/RT)**		**surgical margins: R1+**		**Positive lymph nodes: N+**	
0–4 pts	3,301	454	13.7%	738	22.4%	971	29.4%	744	22.5%
5–8 pts	906	160	17.6%	354	39.1%	424	46.7%	346	38.2%
9–12 pts	518	128	24.7%	253	48.9%	292	56.4%	260	50.2%
>12 pts	104	35	33.6%	62	59.6%	71	68.3%	62	59.6%

*Stratified by model subgroups*.

This model was created as a tool to aid the clinician in estimating the CSS within the heterogeneous high-risk PCa group. It is able to distinguish those who will fare well from those who will benefit poorly from RP, irrespective of future need for adjuvant therapy. It can thus help tilt the scale toward more or less intense treatment based upon more detailed high-risk patient and tumor characteristics.

Remarkably, the lowest score category (0–4 pts) makes up a very significant part of the entire cohort (*n* = 3,186; 65.3%). This implies that practitioners are already intuitively capable of selecting the best from the worst within the high-risk Pca group. This selection bias is a major explanation for the favorable 5- and 10-years CSS of the general high-risk PCa cohort.

The magnitude of this international multi-center patient cohort is undoubtable the major strength of this study. Compromising more than 20 years of interinstitutional data collection, each center treated patients according to their own protocol and standards. This presents a more realistic reflection of general population and practice. Secondly, this subcategorization of established preoperative high-risk factors enables a more accurate prediction of CSS after RP, thus helping to identify those with good prospects after surgery. Thirdly, by using ISUP grading we follow the new pathological classification. Finally, the model is made clinically accessible through an easy-to-use online calculator.

This study is however not without limitations. Firstly, a retrospective study has inherent limitations due to variable data quality. Secondly, the EMPaCT database consists only of men treated by RP, thus a selection bias of fit men is inevitable. thirdly, no data was available concerning the number of positive cores in biopsy samples, as suggested by Sundi et al. Finally, interinstitutional variability impedes standardization.

## Conclusion

By subdividing the established preoperative high-risk factors for prostate cancer, a new model is presented. The extended stratification provides a more accurate prediction of CSS after radical prostatectomy for non-metastatic high-risk prostate cancer. A free online calculator is offered to simplify clinical use.

## Data Availability Statement

The datasets generated for this study are available on request to the corresponding author.

## Ethics Statement

This study was approved by the Ethical Committee of the University Hospitals Leuven.

## Author Contributions

BC conducted the literature research, statistics, data gathering and processing, and writing. SJ, WE, MA, GM, KH, and HVP provided critical feedback. GD data gathering. LM and FC contributed to the data and statistics. PG, RK, CG, PC, MG, BK, GM, RS, BT, HVDP, JW, MS, and AB contributed to the EMPaCT database.

### Conflict of Interest

The authors declare that the research was conducted in the absence of any commercial or financial relationships that could be construed as a potential conflict of interest.
